# Cervical Angina as a Cause of Non-Cardiac Chest Pain: A Case Report

**DOI:** 10.7759/cureus.36279

**Published:** 2023-03-17

**Authors:** Taku Harada, Mori Nakai

**Affiliations:** 1 General Medicine, Nerima Hikarigaoka Hospital, Tokyo, JPN; 2 Diagnostic Generalist Medicine, Dokkyo Medical University Hospital, Mibu, JPN

**Keywords:** internal medicine (general medicine), anginal chest pain, atypical chest pain, non-cardiac chest pain, cervical angina

## Abstract

Cervical angina is a form of non-cardiac chest pain that originates in the cervical spine or cervical cord; it is an under-recognized and easily underdiagnosed condition. Patients with cervical angina often report delayed diagnosis. Here, we report the case of a 62-year-old woman with a history of cervical spondylosis and undiagnosed recurrent chest pain who presented with numbness in the left upper arm and was diagnosed with cervical angina. Although most cases of cervical angina involve uncommon self-limited diseases that improve with conservative treatment, timely diagnosis can reduce patient anxiety and unnecessary office visits and tests. The critical aspect of chest pain evaluation is to rule out fatal disease. Once fatal disease is ruled out, cervical angina should be considered in differential diagnosis if there is a history of cervical spine disease, if the pain radiates to the arm, if it is elicited by cervical spine range of motion or upper extremity movement, or if the chest pain lasts less than a few seconds.

## Introduction

Cervical angina was first described by Nachlas in 1934 [1]. It is a chest pain syndrome involving the medial and lateral pectoral nerves, caused mainly by nerve root compression (cervical or ventral), disorders of the cervical spine, or damage to the cervical cord [2-4]. The exact prevalence remains unknown; however, the estimated prevalence rates are 1.4% in patients with a history of cervical spine surgery [5] and 5% in patients with C7 radiculopathy. Consequently, cervical angina is underdiagnosed owing to the varying prevalence rates [6,7].

Nerve root compression appears at C5-C6 (37%), C6-C7 (30%), C4-C5 (27%), and C3-C4 (4%) [2,3]. Cervical angina often develops anterior chest pain that is explained as sharp, aching, tightness, or squeezing. Some patients may be mitigated with nitroglycerin [3]. Symptoms may appear at rest or worsen with exercise. Chest pain may be accompanied by headache, neck pain, shoulder pain, and arm pain. Most patients with cervical angina from cervical radiculopathy respond well to conservative treatments. However, surgery may be the treatment of choice in refractory cases [2,3].

Cervical angina is listed as one of the causes of non-cardiac chest pain (NCCP) [3,8]. The diagnosis of cervical angina remains under-recognized [3,5]; similar to patients with other causes of NCCP, many patients with cervical angina undergo extensive cardiac evaluation and often report delayed diagnosis [3,6]. Cervical angina is one of the causes of NCCP, and these patients share this extended diagnostic experience with patients suffering other causes. In this case, a 62-year-old woman who had a history of cervical spondylosis and undiagnosed recurrent chest pain was diagnosed with cervical angina. This case highlights the importance of diagnosis of cervical angina, even if it is a non-critical self-limiting condition.

## Case presentation

A 62-year-old woman presented to the clinic with chest discomfort and pain that developed a few days earlier. Her underlying medical conditions included Sjögren syndrome, hypertension, dyslipidemia, depression, panic disorder, and a history of undiagnosed recurrent chest pain lasting almost five years. One month before the visit, she had been experiencing occasional numbness extending from the left forearm to the outer side of the upper arm. A few days before the visit, she became aware of left-sided chest pain, which developed without any particular trigger, including during rest, and appeared frequently during the day, lasting for less than 1 min. The chest pain extended laterally from the left anterior chest to the lateral chest, was localized to an area larger than the size of the palm and did not extend beyond the midline. She had no history of trauma or heavy lifting.

There was no alteration in the pain intensity on deep breathing or changing posture. Moreover, the patient reported no abdominal pain, burping, or heartburn. The patient had a history of depression and panic disorder and expressed strong anxiety regarding the undiagnosed recurrent chest pain with an uncertain prognosis.

Physical examination revealed negative test results for the Jackson maneuver and Spurling maneuver, normal upper limb tendon reflexes, and normal Hoffman and Tremner test results. The results of the high-sensitivity troponin I assay were 0.010 ng/ml (negative) and electrocardiogram findings were also normal. After an acute coronary syndrome was considered unlikely, medical information including medical history, laboratory findings, and radiographic information was reviewed in detail, including evaluation for undiagnosed recurrent chest pain. Previous MRI scan findings recorded one year earlier revealed intervertebral foramen stenosis at C4/5 and C6/7, indicating cervical spondylosis. The patient did not remember being diagnosed with cervical spondylosis in the past. With the assistance of past imaging results, we explained the possibility of cervical angina and that most cases would improve with conservative treatment within three months. Therefore, an outpatient follow-up was suggested during which the patient reported alleviation of symptoms. After approximately one month, the symptoms resolved completely.

## Discussion

We encountered a case of cervical angina in a 62-year-old woman with a history of cervical spondylosis and undiagnosed chest pain, which occurred frequently. Even in the case of a non-critical self-limiting uncommon condition, physicians have a responsibility to make a timely and accurate diagnosis in order to alleviate a patient’s anxiety and prevent unnecessary visits, tests, and examinations [9-11]. The first step in evaluating chest pain is to rule out fatal disease. However, once this is done, it is important to consider cervical angina in the differential if there is a history of cervical spine disease, if the pain radiates to the arm, if it is provoked by cervical spine range of motion or upper extremity movement, or if the chest pain lasts for less than a few seconds.

Possible reasons for the difficulty in diagnosing cervical angina include difficulty in recognition, the confusing nature of the disease itself, and anatomical complexities. Although cervical angina was first reported in 1934 [1], the diagnosis of cervical angina is still not well recognized [3,5]. In many cases, neurological symptoms are neglected [4]. In addition, 50-60% of patients experience autonomic symptoms (dyspnea, dizziness, nausea, sweating, pallor, fatigue, diplopia, and headache), the mechanisms of which are not well understood [2,3,5]. In addition, the fact that pain symptoms resemble angina pectoris and have been reported to be relieved by nitroglycerin [3] may also be a predisposing factor to impede the diagnosis. It has been reported that many patients with cervical angina undergo various cardiac evaluations, often with delayed diagnosis [3,6].

Cervical angina also encompasses diagnostic difficulties in terms of anatomy. First, it has been reported that cervical radiculopathy often deviates from its anatomic distribution. A study by Rainville et al. [12] showed overlapping symptoms of C6 and C7 radiculopathy, and a recent autopsy study by Guday et al. [13] found that anatomic variations in the brachial plexus were present in 25% of the reported cases. In addition, physical findings useful in the examination of cervical angina are not yet known, which may be attributed to the related diagnostic difficulties. Spurling maneuver, a physical examination in which the head is compressed downward while the cervical spine is rotated to the symptomatic side, has been reported to reproduce the symptoms of cervical angina [2], but reports also suggest that neurological findings are often absent or nonlocalized in cervical angina [2,3], and there is no consistency. In the present case, the Spurling test was negative, but this may be related to the fact that the pressure drainage was mild and the numbness in the chest and upper extremities had diminished at the time of examination.

Cervical angina is an NCCP [3,8], but the diagnosis of NCCP itself is often neglected: only half of the patients with NCCP are referred to other departments after cardiac causes have been ruled out [14], and many patients do not have a confirmed diagnosis and receive inappropriate treatments [15]. Moreover, patients with NCCP often seek medical attention for recurrent and persistent chest pain [8,16], and are mainly anxious about their symptoms and the possibility of serious illness [17], often exacerbating psychiatric disorders and comorbidities, resulting in decreased quality of life [8]. Accurate diagnosis of NCCP not only prevents unnecessary clinic visitations and tests, but it can help improve the patient's quality of life, and help reduce stigmatization of patients diagnosed with "psychogenic" illnesses.

The differential diagnoses in this case other than NCCP may include angina pectoris, microvascular angina, precordial catch syndrome, or psychogenic cardiac illness.

Angina pectoris is characterized by recurrent chest pain that is triggered or exacerbated by exercise, anxiety, or stress, and is relieved within minutes by nitroglycerin administration or rest [18]. Unlike angina, microvascular angina has angina-like symptoms despite having normal coronary arteries on angiography. In the past, the name "cardiac syndrome X" was used, as the pathophysiology was unknown. Myocardial ischemia is now thought to be caused by microvascular dysfunction and is clinically classified into four subtypes according to its underlying etiology [19]. The duration of chest pain in microvascular angina has been reported to last longer than in angina, and the time from rest or use of nitroglycerin to disappearance of chest pain is 10-15 minutes, which is longer than in angina [20,21]. Based on the information above, we do not believe our case has microvascular angina as the duration of chest pain was very short and there were no laboratory findings suggestive of ischemia during chest pain. Precordial catch syndrome is a disorder of musculoskeletal chest pain that causes short-lived pain lasting a few seconds and can be episodic. Although precordial catch syndrome can occur at any age, the typical clinical course occurs primarily in children and generally lasts between 30 seconds to 3 minutes [22]. It is exacerbated by deep breathing and is limited to 1-2 finger widths, which does not match this case [22]. One of the criteria for determining whether chronic pain is psychogenic or derived from a medical disorder is A-MUPS [23]. In line with this criterion, our patient had a history of mental disorder and an unclear provocative/palliative factor. However, the lack of persistence without cessation of pain and stressful episodes suggests an organic disease and, in addition, careful exclusion is essential when diagnosing psychogenic cardiac illness. Therefore, it is necessary to explore all possible organic diagnoses before reaching this diagnosis.

To timely diagnose cervical angina, Feng et al. [4] argue that it is necessary to recognize chest pain which is associated with cervical angina, and to be suspicious of patients who complain of chest pain for which the diagnosis is uncertain [4]. Moreover, cervical imaging could be an important diagnostic modality in cervical angina, once coronary artery disease has been adequately ruled out [4]. Thus, a history of cervical spine disease and cervical spine imaging, if available, should be confirmed. In addition to the presence of relevant history, clinical features of cervical angina include pain elicited by cervical spine range of motion or upper extremity movement, a recent history of physical exertion (lifting, pulling, pushing, such as yard work, or lifting heavy objects), and pain lasting for more than 30 min or less than 5 s; a positive Spurling maneuver examination result and the presence of neurologic symptoms associated with specific skin or muscle segments [3] may also be suggestive. Radiographs and MRI findings may show degenerative changes in the cervical spine, such as disc stenosis, osteophyte formation, and neuroforaminal invasion. MRI can also rule out spinal changes, fractures, tumors, and infections that require urgent intervention. Additionally, electromyography could also support the diagnosis [3].

## Conclusions

Even in non-critical, self-limiting, uncommon conditions, timely and accurate diagnosis is important for physicians to reduce patient anxiety and prevent unnecessary visits and tests. It is important to rule out a fatal disease, but it is also important to include cervical angina in the differential if there is a history of cervical spine disease or if the NCCP radiates to the arm or is induced by cervical spine range of motion or upper extremity movement.
